# Oral healthcare providers play a vital role in vaccination efforts: Patient perspectives

**DOI:** 10.1002/cre2.777

**Published:** 2023-10-06

**Authors:** Sara Steinbaum, Julia Jagannath, Lake Seymour, Patricia Corby, Roopali Kulkarni, Katherine France

**Affiliations:** ^1^ Department of Orthodontics University of Pennsylvania School of Dental Medicine Philadelphia Pennsylvania USA; ^2^ Private Practice Blue Bell Pennsylvania USA; ^3^ Department of Orthodontics Harvard School of Dental Medicine Boston Massachusetts USA; ^4^ Department of Oral Medicine University of Pennsylvania School of Dental Medicine Philadelphia Pennsylvania USA

**Keywords:** coronavirus disease 2019 (COVID‐19), human papillomavirus (HPV), vaccine advocacy, vaccines

## Abstract

**Objectives:**

Human papillomavirus (HPV) is associated with 70% of oropharyngeal squamous cell carcinomas. Coronavirus disease 2019 (COVID‐19) is the infectious cause of a global pandemic that killed millions worldwide. Effective vaccinations exist against both diseases, but patient acceptance remains a challenge. The objective of this study was to assess patients' attitudes toward oral healthcare providers' (OHCPs) roles in HPV and COVID‐19 vaccinations.

**Methods:**

A cross‐sectional survey of young adult patients was distributed in Philadelphia, PA, between April and June 2021. The survey assessed knowledge and attitudes around OHCPs serving various roles in COVID‐19 and HPV vaccination.

**Results:**

Nearly 70% of 163 respondents would accept the recommendation for a COVID‐19 vaccine from an OHCP, while 56% would for HPV. Those previously vaccinated against COVID‐19 were more comfortable discussing COVID‐19 vaccines (92%, *p* < .001) or HPV vaccines (76%, *p* < .001) with OHCPs compared to those who were unvaccinated against COVID‐19. African American/Black patients were less comfortable discussing vaccines, irrespective of vaccination status.

**Conclusions:**

OHCP can play a vital role in increasing the overall COVID‐19 and HPV vaccination status of the public, as demonstrated by the high acceptance of dental involvement in both vaccine campaigns. Racial disparity in vaccination attitude is a public health challenge that needs to be addressed.

## 1 INTRODUCTION

Human papillomavirus (HPV) is the most common sexually transmitted infection in the United States (US) (Centers for Disease Control and Prevention, 2022). About 30% of all head and neck cancers are related to HPV, and about 70% of oropharyngeal squamous cell carcinomas (OPSCCs) are caused by HPV (How Many Cancers, 2021b). Furthermore, the incidence of HPV‐related OPSCC is increasing and has surpassed the rates of HPV‐related cervical cancers (Anantharaman et al., 2017). Vaccines against HPV (distributed as Gardasil, among others) have been available since 2006, are highly effective, and are currently recommended for adolescents of all genders starting at age 11–12. Catch‐up doses are recommended for individuals under the age of 26 and selected adults aged 26–45 years. In June 2020, the US Food and Drug Administration (FDA) expanded the approved uses of the Gardasil‐9 (nonavalent) vaccine to include prevention of OPSCCs and other head and neck cancers caused by HPV (U.S. Food and Drug Administration, 2020).

The coronavirus disease 2019 (COVID‐19) pandemic has significantly impacted healthcare systems worldwide (WHO Health Emergency Dashboard, 2022). The urgent development of vaccines against COVID‐19 led to a call for healthcare workers, including OHCPs, to help increase distribution of COVID‐19 vaccines (Centers for Disease Control and Prevention, 2021a; The White House, 2021; U.S. Food and Drug Administration, 2022). The advent and spread of COVID‐19 vaccines, which can significantly reduce the risk of severe illness and death after infection, has also expanded discussion of healthcare provider roles in vaccinations and the ability of oral healthcare providers (OHCPs) to aid in preventive care.

Before the COVID‐19 pandemic, few states had legislation granting OHCPs the ability to administer vaccines (American Dental Association, 2022). Support for incorporating vaccine promotion and administration into the dentist's scope of practice includes their experience with administering local anesthesia, higher frequency of patient appointments compared to that of primary care physicians, and medical expertise (Smith & Smith, 2017). Previous studies exploring the attitudes of OHCPs administering vaccines have shown mixed results. Patel et al. (2020) demonstrated that dentists are willing to receive training on recommending vaccines. However, an American Dental Association (ADA) Clinical Evaluators Panel survey revealed that 38% of responding dentists would be uncomfortable administering the HPV vaccine (Patton et al., 2020). In a series of studies, Daley and colleagues have found that dentists have mixed readiness to discuss vaccines. In addition, patients are more receptive to vaccine and risk discussions with dentists compared to allied dental health professionals and to receiving information from same‐gender providers (Daley et al., 2014, 2021). Surveys by both Dean et al. (2020) and Stull et al. (2020) showed that a majority of parents were receptive to receiving HPV‐related information from dental professionals and would allow their child to receive the HPV vaccine from their dentist, while a study by Lazalde et al. (2018) found that only one‐quarter of parents were comfortable with dentists administering the HPV vaccine. Differing results for dentist‐administered vaccines from both the dentist and patient perspective call for further research on this subject.

Vaccinations against both COVID‐19 and HPV have been administered to millions of individuals in the United States. Although the two vaccines address different public health concerns, increased vaccinations against both are essential to improve health outcomes. In addition, the COVID‐19 pandemic has caused a decrease in delivery of routine vaccinations, including for HPV (Healthy People, 2020; Saxena et al., 2021). In October 2020, the ADA expressed support for dentists administering vaccines via Resolution 91H‐2020 (Solana, 2020). The majority of states have allowed dentists to administer COVID‐19 vaccines, although Pennsylvania, where this survey was distributed, has not allowed either COVID‐19 or HPV vaccination by OHCPs to date (American Dental Association, 2022).

The aim of this study was to assess patients' attitudes regarding OHCPs' roles in HPV and COVID‐19 vaccinations from education to administration. To our knowledge, this is the first cross‐sectional study that investigates the role of any health professional in vaccination against COVID‐19 and HPV and the first to analyze patients' attitudes relating to OHCPs' roles in vaccines for COVID‐19. Understanding differences in patients' attitudes between vaccines could aid in patient education and may provide helpful background on distribution strategies.

## 2 METHODS

A cross‐sectional survey was administered to a convenience sample of young adult patients (ages 18–45) at an academic dental institution in Pennsylvania from April to June 2021. The survey assessed attitudes and acceptance of receiving COVID‐19 and HPV vaccinations in general and when administered by dental providers. A previously published questionnaire regarding patient acceptance of HPV vaccination by dental providers was adapted to apply to an adult population and to inquire about both HPV and COVID‐19 (Dean et al., 2020). The adapted survey included questions about patients' attitudes and acceptance of COVID‐19 and HPV vaccination, perceptions of dental care providers' role in COVID‐19 and HPV vaccination, and comfort in having a dental provider administer COVID‐19 and HPV vaccines (Table 1). Despite mixed evidence of acceptance from nondentist providers in the dental setting (Daley et al., 2021), the term “dental provider” was used throughout the survey due to wide comprehensibility and to maximize inclusivity.

**Table 1:** List of survey questions and respondents' answers showing comfort with dental provider involvement in vaccine efforts around both the COVID‐19 and HPV vaccines.

COVID‐19 Questions	Yes	No	Unsure
1. Have you been completely vaccinated against COVID‐19?	90 (55%)	73 (45%)	
2. If no, do you plan on receiving the COVID‐19 vaccine(s)?	31 (19%)	18 (11%)	23 (14%)
3. Have you been informed that the COVID‐19 vaccine can prevent severe illness caused by COVID‐19?	151 (93%)	4 (3%)	8 (5%)
4. Do you believe dental professionals are qualified to educate you about the COVID‐19 vaccines available?	99 (61%)	19 (12%)	44 (27%)
5. Would you feel comfortable discussing COVID‐19 vaccines with your dental provider?	127 (78%)	22 (13%)	14 (9%)
6. Would you accept the recommendation for a COVID‐19 vaccine from your dental provider?	109 (67%)	26 (16%)	28 (17%)
7. Would you feel comfortable allowing a dental provider to administer a COVID‐19 vaccine for you?	87 (53%)	44 (27%)	31 (19%)
	**Accept vaccine**	**Refuse vaccine**	**Not applicable** (i.e., already vaccinated or awaiting a scheduled vaccination appointment)
8. If your dental provider offered you a COVID‐19 vaccine today, you would likely:	52 (32%)	35 (21%)	74 (45%)

**HPV Questions**	Yes	No	Unsure
1. Have you been completely vaccinated against human papillomavirus (HPV)?	83 (51%)	77 (47%)	
2. If no, do you plan on receiving the human papillomavirus (HPV) vaccine series?	13 (8%)	29 (18%)	35 (22%)
3. Have you been informed that the human papillomavirus (HPV) vaccine can prevent some types of head and neck cancer?	81 (50%)	58 (36%)	20 (12%)
4. Do you believe dental professionals are qualified to educate you about the human papillomavirus (HPV) vaccines available?	85 (52%)	23 (14%)	54 (33%)
5. Would you feel comfortable discussing human papillomavirus (HPV) vaccines with your dental provider?	102 (63%)	33 (20%)	27 (17%)
6. Would you accept the recommendation for a human papillomavirus (HPV) vaccine from your dental provider?	91 (56%)	38 (23%)	33 (20%)
7. Would you feel comfortable allowing a dental provider to administer a human papillomavirus (HPV) vaccine for you?	80 (49%)	45 (28%)	36 (22%)
	**Accept vaccine**	**Refuse vaccine**	**Not applicable** (i.e., already vaccinated or awaiting a scheduled vaccination appointment)
8. If your dental provider offered you a human papillomavirus (HPV) vaccine today, you would likely:	55 (34%)	57 (35%)	50 (31%)

*Note*: Some percentages may not sum to 100% due to missing responses and rounding.

The survey was distributed when all COVID‐19 vaccines in the United States were authorized for Emergency Use. Researchers were calibrated on patient eligibility and survey distribution and used a standardized script to ask if patients would like to participate. Patients were excluded if they were unable to read or respond to the survey in English without a translator, lacked medical decision‐making capacity, or were younger than 18 or older than 45.

Verbal informed consent was obtained from each patient, and survey details, including risks and benefits of participating, were provided before starting. The surveys were administered on iPads using the Research Electronic Data Capture (REDCap) secure system in the waiting rooms of the academic dental center (Harris et al., 2009). The study was approved by the local Institutional Review Board as exempt.

Before starting the survey, participants were given background information about both COVID‐19 and HPV to review. Patients were allowed unlimited time to complete the survey without any assistance. Participants were given the opportunity to enter their email address into a raffle for the chance to win a $100 gift card after completing the survey. This data was not counted as part of submitting a completed survey, and collected emails were not linked to survey responses. Raffle prize distribution was the only follow‐up communication after the survey was completed.

Power calculation was completed accounting for 50%–80% acceptance of various roles in vaccination efforts around HPV reported in previous studies and no current estimate of patient acceptance of OHCP roles in COVID‐19 vaccination efforts (Dean et al., 2020; Stull et al., 2020). Sample size for this study was estimated as 158 (80% power, *α* = .05, *β* = .20). This estimate assumed that each patient would only fill out questions about one vaccine (COVID‐19 or HPV), leaving the other questions blank. Given that all patients were asked to fill out the survey in its entirety, including questions about both diseases, this sample represented a conservative estimate.

Data were analyzed using R software (R Foundation for Statistical Computing) and Microsoft Excel (Microsoft Corporation). Data were collected on sex assigned at birth, gender identity, age, race, education, household income, vaccination status and plans, education about the vaccines, comfort, and attitudes about the dental provider's role in vaccinations for both COVID‐19 and HPV. For analysis, we simplified age and race into 3‐level categorical variables (younger than 24, ages 24–40, and 40 and older and White, African American/Black, and others, respectively). We simplified education level into less than high school, some college, bachelor's degree, and graduate degrees. When analyzing responses for whether patients would be comfortable receiving a vaccine today (HPV or COVID‐19), we removed individuals already vaccinated from analysis of this question only. We completed descriptive statistics around demographic variables and survey answers. We compared survey answers according to demographic information and vaccination status for both diseases. We evaluated the significance for each using *χ* ^2^ tests and Fisher's exact tests. For demographic information, we also compared answers across the COVID‐19 and HPV vaccines using multivariate analysis of variance. Significance for all comparisons was set at *p* < .1 and further stratified using the significance scale of *p* < .01***, 0.01<*p* < .05**, and 0.05 < *p* < .1*. The current data and associated analyses were considered exploratory, with additional validation and survey distribution planned as next steps.

## 3 RESULTS

Of the 163 patients who completed the survey, the majority of participants were female (67%) and the mean age was 31.6 (standard deviation 7.67, range 18–45). Over 60% of survey participants identified with a racial minority group, with the largest group identifying as African American/Black (72 participants, 44%) (Table 2). White patients accounted for 32% of our sample (52 patients), while other racial groups were far less frequently represented. For strength of statistical analysis, given this imbalance and small representation of certain groups (i.e., American Indian/Alaska Native, *n* = 1 patient), racial groups outside of African American/Black and White patients were collapsed and compared as a single group. This includes patients of multiple races. About one‐half of participants were vaccinated against each of COVID‐19 (55%) and HPV (51%) (Table 2). Of those not currently vaccinated, 19% were planning to get the COVID‐19 vaccines and 8% were planning to get the HPV vaccines. The vast majority (93%) of respondents had been previously informed that the COVID‐19 vaccines can prevent severe illness caused by COVID‐19. However, only 50% of respondents had been previously informed that the HPV vaccines can prevent some forms of head and neck cancer (Table 1).

**Table 2:** Demographic information of survey respondents.

Variable	Statistic
Gender	
Female	109 (67%)
Age	
Count	158
Mean (SD)	31.61 (7.67)
Race^a^	
White	52 (32%)
African American/Black	72 (44%)
Hispanic/Latin	12 (7%)
American Indian/Alaska Native	1 (1%)
Asian	16 (10%)
Prefer not to say/other	10 (6%)
Education	
≤High School	43 (26%)
Some College	44 (27%)
Bachelor	51 (31%)
Graduate	25 (15%)
Household income (k)	
<20	50 (31%)
20–35	28 (17%)
35–50	25 (15%)
50–75	24 (15%)
75–100	19 (12%)
>100	16 (10%)

^a^ Percentages sum to over 100% as patients were asked to select all categories that apply.

**Figure 1:**
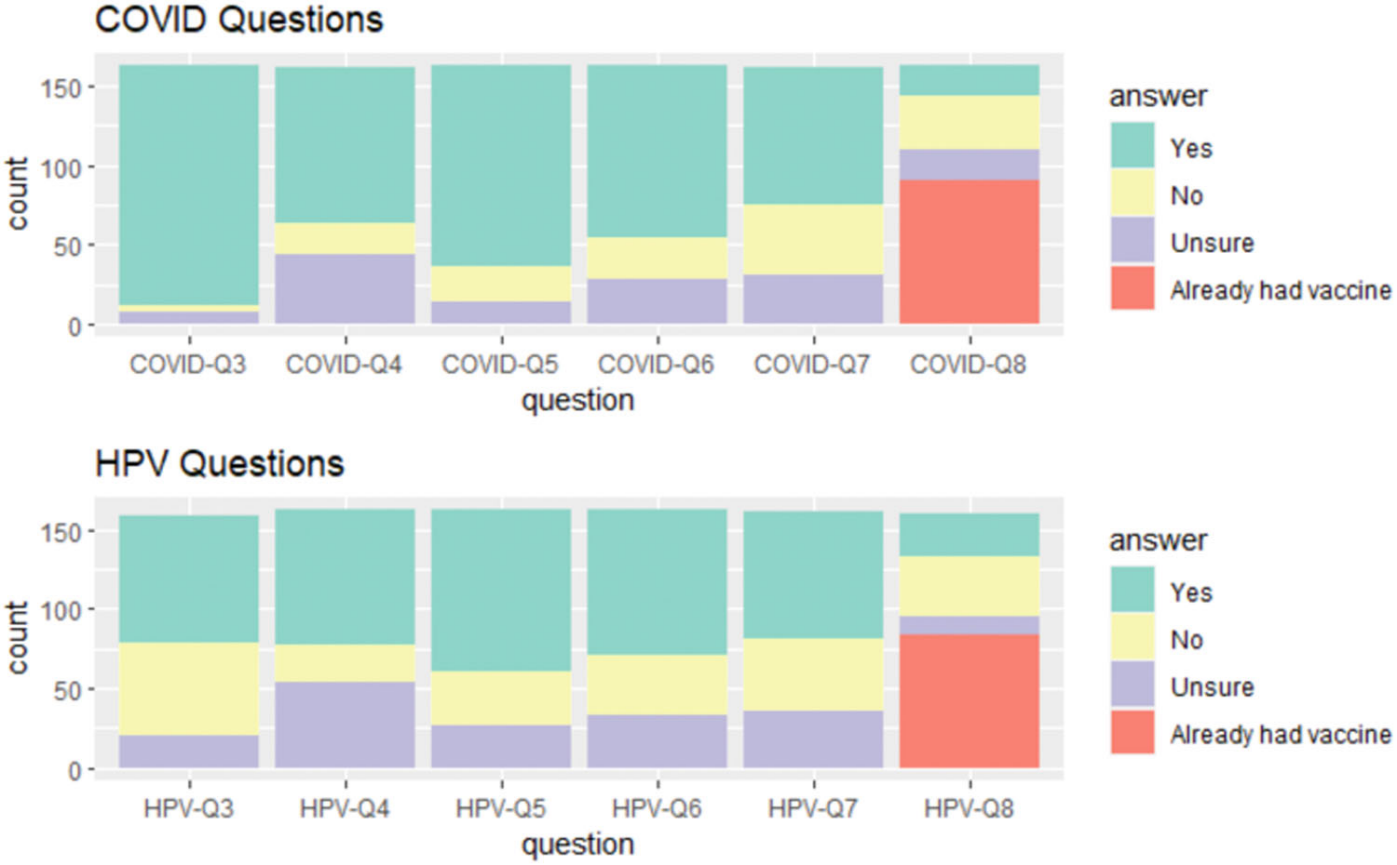
Graphic representation survey response distribution by question, divided into COVID‐19 and HPV categories. “COVID‐Q3” refers to whether respondents were aware that COVID‐19 vaccines can prevent severe illness caused by COVID‐19. “HPV‐Q3” refers to whether respondents were aware that HPV vaccines can prevent some forms of head and neck cancers. “COVID‐Q4” and “HPV‐Q4” refer to whether respondents believed dental providers are qualified to educate about each vaccine. “COVID‐Q5” and “HPV‐Q5” refer to whether respondents feel comfortable discussing each vaccine with their dental provider. “COVID‐Q6” and “HPV‐Q6” refer to whether respondents would accept a recommendation for each vaccine from their dental provider. “COVID‐Q7” and “HPV‐Q7” refer to whether respondents would feel comfortable allowing a dental provider to administer each vaccine. “COVID‐Q8” and “HPV‐Q8” refer to whether respondents would accept each vaccine if offered today.

The majority of respondents believed that dental providers are qualified to educate about the COVID‐19 (61%) and the HPV vaccines (52%). A large proportion of survey respondents reported feeling comfortable discussing the COVID‐19 vaccines with dental providers (78%), while a smaller majority felt comfortable discussing the HPV vaccines (63%). Nearly 70% of respondents would accept the recommendation for a COVID‐19 vaccine (67%) from a dental professional, compared to fewer for HPV (56%). About one‐half of respondents felt comfortable allowing a dental professional to administer the COVID‐19 (53%) or HPV (49%) vaccines. About one‐third of respondents would accept a COVID‐19 (32%) versus HPV vaccine (34%) from a dental provider today if offered (Table 1). The distributions of survey responses can be visualized in Figure 1. None of these direct findings were significantly different when comparing responses about the COVID‐19 and HPV vaccines.

Those previously vaccinated against COVID‐19 were more receptive to dental provider involvement in vaccine initiatives (Table 3). A higher proportion of those vaccinated against COVID‐19 believed that dental providers were qualified to educate about the COVID‐19 vaccines (69%) compared to those not vaccinated (51%, *p* = .074). They were also more likely to believe that dental providers were qualified to educate about the HPV vaccines (64%, *p* = .0013). These participants were more likely to be comfortable discussing the COVID‐19 vaccines (92%, *p* < .0001) or HPV vaccines (75%, *p* = .0003) with their dental providers than those who were unvaccinated against COVID‐19. A greater percentage would accept a recommendation for a COVID‐19 (82%, *p* < .0001) or HPV vaccine (67%, *p* = .0006) from a dental provider when compared to those not vaccinated against COVID‐19. Those vaccinated against COVID‐19 were more comfortable with dental providers administering COVID‐19 (66%, *p* = .0011) or HPV (63%, *p* = .0001) vaccines. Similar trends existed for the group of respondents who were planning to receive a COVID‐19 vaccine. A higher proportion of individuals planning to receive a COVID‐19 vaccine were comfortable with dental providers administering COVID‐19 (65%, *p* < .0001) and HPV (60%, *p* < .0001) vaccines. HPV vaccination status did not result in the same stratification, although patients were generally comfortable discussing the COVID‐19 or HPV vaccines with dental providers.

**Table 3:** Survey responses according to COVID‐19 vaccination status of participants showing count and percentage for responses to survey questions about dental provider roles in vaccination for COVID‐19 and HPV, and comparing responses across vaccinations.

		Yes	No COVID (%) | HPV (%)	Unsure	*p*‐Value^a^ COVID | HPV	*p*‐Value^b^ (COVID vs. HPV)
Dental providers are qualified to educate about vaccines						
COVID vaccine	Yes	62 (69%) | 58 (64%)	8 (9%) | 7 (8%)	20 (22%) | 25 (28%)	0.074*^,^ c | 0.0013***	0.015**
	No	37 (51%) | 27 (38%)	11 (15%) | 16 (22%)	24 (33%) | 29 (40%)		
Plan for COVID vaccine	Yes	21 (68%) | 15 (50%)	2 (6%) | 4 (13%)	8 (26%) | 11 (37%)	0.019** | 0.12	
	No	7 (41%) | 6 (33%)	6 (35%) | 7 (39%)	4 (24%) | 5 (28%)		
	Unsure	8 (35%) | 6 (26%)	3 (13%) | 4 (17%)	12 (52%) | 13 (57%)		
Comfortable discussing vaccines with dental providers						
COVID vaccine	Yes	83 (92%) | 68 (76%)	2 (2%) | 910%)	5 (6%) | 13 (14%)	<0.0001*** | 0.0003***	0.0002***
	No	44 (60%) | 34 (47%)	20 (27%) | 24 (33%)	9 (12%) | 14 (19%)		
Plan for COVID vaccine	Yes	25 (81%) | 18 (60%)	5 (16%) | 8 (27%)	1 (3%) | 4 (13%)		
	No	8 (44%) | 6 (33%)	10 (56%) | 11 (61%)	0|1 (6%)		
	Unsure	11 (48%) | 10 (43%)	4 (17%) | 4 (17%)	8 (35%) | 13 (57%)		
Accept Vaccine Recommendations from Dental Providers						
COVID vaccine	Yes	74 (82%) | 60 (67%)	8 (9%) | 11 (12%)	8 (9%) | 19 (21%)	<0.0001*** | 0.0006***	0.0001***
	No	35 (48%) | 31 (43%)	18 (25%) | 27 (38%)	20 (27%) | 14 (19%)		
Plan for COVID vaccine	Yes	26 (84%) | 21 (70%)	3 (10%) | 5 (17%)	2 (6%) | 4 (13%)	<0.0001*** | 0.0001***	<0.0001***
	No	1 (6%) | 2 (11%)	12 (67%) | 14 (78%)	5 (28%) | 2 (11%)		
	Unsure	7 (30%) | 8 (35%)	3 (13%) | 7 (30%)	13 (57%) | 8 (35%)		
Comfortable with Dental Providers Administering Vaccines						
COVID vaccine	Yes	59 (66%) | 56 (63%)	15 (17%) | 14 (16%)	16 (18%) | 19 (21%)	0.0011*** | 0.0001***	0.033**
	No	28 (39%) | 24 (33%)	29 (40%) | 31 (43%)	15 (21%) | 19 (24%)		
Plan for COVID vaccine	Yes	20 (65%) | 18 (60%)	4 (13%) | 6 (20%)	7 (23%) | 6 (20%)	<0.0001***|<0.0001***	0.044**
	No	2 (11%) | 1 (6%)	15 (83%) | 15 (83%)	1 (6%) | 2 (11%)		
	Unsure	5 (23%) | 5 (22%)	10 (45%) | 9 (39%)	7 (32%) | 9 (39%)		

Abbreviation: MANOVA, multivariate analysis of variance.

a Statistical tests for each vaccine: Chi‐squared/Fisher's exact test.

b Statistical tests between vaccines: MANOVA test.

c *p*‐Value significance code: *p* < .01***, 0.01 < *p* < .05**, 0.05 < *p* < .1*.

The majority of those planning to get an HPV vaccine (62%, *p* = .0148) and of those unsure if planning to get an HPV vaccine (57%, *p* = .015) believed that dental providers are qualified to educate about HPV vaccines (Table 4). Over 70% (*p* = .0082) of those planning to and of those unsure whether to get an HPV vaccine were comfortable discussing the vaccines with dental providers. Comparatively, of those planning to receive an HPV vaccine, 77% would accept the recommendation from a dental provider for the HPV vaccines and 66% of those unsure would accept the recommendation. The majority of those planning to receive an HPV vaccine would be comfortable with dental providers administering the HPV (75%, *p* < .0001) and COVID‐19 vaccines (62%, *p* = .0055).

**Table 4:** Survey responses according to HPV vaccination status of participants showing count and percentage for responses to survey questions about dental provider roles in vaccination for COVID‐19 and HPV, and comparing responses across vaccinations.

		Yes	No COVID (%) | HPV (%)	Unsure	*p*‐Value^a^ COVID|HPV	*p*‐Value^b^ (COVID vs. HPV)
Dental providers are qualified to educate about vaccines						
HPV vaccine	Yes	51 (61%) | 43 (52%)	8 (10%) | 9 (11%)	24 (29%) | 31 (37%)	0.64 | 0.30	0.73
	No	45 (59%) | 41 (53%)	11 (14%) | 14 (28%)	20 (26%) | 22 (29%)		
Plan for HPV vaccine	Yes	7 (54%) | 8 (62%)	1 (8%) | 1 (8%)	5 (38%) | 4 (31%)	0.12 | 0.015**^,^ c	0.69
	No	16 (55%) | 13 (45%)	8 (28%) | 11 (38%)	5 (17%) | 5 (17%)		
	Unsure	22 (65%) | 20 (57%)	2 (6%) | 2 (6%)	10 (29%) | 13 (37%)		
Comfortable discussing vaccines with dental providers						
HPV vaccine	Yes	66 (80%) | 54 (65%)	12 (14%) | 16 (19%)	5 (6%) | 13 (16%)	0.45 | 0.86	0.60
	No	58 (75) | 47 (61%)	10 (13%) | 17 (22%)	9 (12%) | 13 (17%)		
Plan for HPV vaccine	Yes	11 (85%) | 10 (77%)	1 (8%) | 1 (8%)	1 (8%) | 2 (15%)	0.28 | 0.0073***	0.66
	No	19 (66%) | 12 (41%)	7 (24%) | 13 (45%)	3 (10%) | 4 (14%)		
	Unsure	28 (80%) | 25 (71%)	2 (6%) | 3 (9%)	5 (14%) | 7 (20%)		
Accept vaccine recommendations from dental providers						
HPV vaccine	Yes	57 (69%) | 51 (61%)	12 (14%) | 19 (23%)	14 (17%) | 13 (16%)	0.81 | 0.29	0.23
	No	50 (65%) | 39 (51%)	14 (18%) | 19 (25%)	13 (17%) | 19 (25%)		
Plan for HPV vaccine	Yes	10 (77%) | 10 (77%)	2 (15%) | 1 (8%)	1 (8%) | 2 (15%)	0.0044** | < 0.0001***	0.23
	No	14 (48%) | 6 (21%)	11 (38%) | 17 (59%)	4 (14%) | 6 (21%)		
	Unsure	26 (74%) | 23 (66%)	1 (3%) | 1 (3%)	8 (23%) | 11 (31%)		
Comfortable with dental providers administering vaccines						
HPV vaccine	Yes	48 (58%) | 45 (54%)	23 (28%) | 26 (31%)	12 (14%) | 12 (14%)	0.30 | 0.056*	0.14
	No	37 (49%) | 34 (45%)	21 (28%) | 19 (25%)	18 (24%) | 23 (30%)		
Plan for HPV vaccine	Yes	8 (62%) | 9 (75%)	2 (15%) | 1 (8%)	3 (23%) | 2 (17%)	0.0055*** | < 0.0001***	0.19
	No	12 (43%) | 9 (31%)	14 (50%) | 16 (55%)	2 (7%) | 4 (14%)		
	Unsure	17 (49%) | 16 (46%)	5 (14%) | 2 (6%)	13 (37%) | 17 (49%)		

Abbreviation: MANOVA, multivariate analysis of variance.

a Statistical tests for each vaccine: Chi‐squared/Fisher's exact test.

b Statistical tests between vaccines: MANOVA test.

c *p‐*Value significance code: *p* < .01***, 0.01 < *p* < .05**, 0.05 < *p* < .1*.

Responses also varied according to race. A greater percentage of White individuals (85%) and those that identified as “Other” (77%) felt comfortable accepting a recommendation for a COVID‐19 vaccine from a dental provider as compared to African American/Black individuals (49%, *p* = .0002). Of note, in the 12 patients who identified as Hispanic but did not identify as African American/Black or White, 82% felt comfortable accepting this recommendation. A similar trend was seen in accepting recommendations for an HPV vaccine from a dental provider (Table 5). Three‐quarters of White individuals were comfortable with dental providers administering the COVID‐19 and HPV vaccines; however, only about one‐third of African American/Black individuals were comfortable. On the other hand, 59% of those from other racial backgrounds reported being comfortable with OHCPs administering the COVID‐19 vaccine and 49% with the HPV vaccine (*p* = .0001 and *p* < .0001, respectively, difference between COVID‐19 and HPV vaccine administration *p* = .0013).

**Table 5:** Survey responses according to participant race showing count and percentage for responses to survey questions about dental provider roles in vaccination for COVID‐19 and HPV, and comparing responses across vaccinations.

	Yes	No COVID (%) | HPV (%)	Unsure	*p*‐Value^a^ COVID | HPV	*p*‐Value^b^ (COVID vs. HPV)
OHCPs are qualified to educate about vaccines					
White	37 (71%) | 30 (58%)	8 (15%) | 8 (15%)	7 (13%) | 14 (27%)	0.059*^,^ c | 0.29	0.080*
African American/Black	39 (55%) | 31 (44%)	6 (8%) | 12 (17%)	26 (37%) | 28 (39%)		
Other	23 (59%) | 24 (62%)	5 (13%) | 3 (8%)	11 (28%) | 12 (31%)		
Comfortable discussing vaccines with OHCPs					
White	45 (87%) | 39 (75%)	4 (8%) | 8 (15%)	3 (6%) | 5 (10%)	0.29 | 0.18	0.19
African American/Black	52 (72%) | 38 (54%)	11 (15%) | 18 (25%)	9 (13%) | 15 (21%)		
Other	30 (77%) | 25 (64%)	7 (18%) | 7 (18%)	2 (5%) | 7 (18%)		
Accept vaccine recommendations from dental providers					
White	44 (85%) | 37 (71%)	5 (10%) | 7 (13%)	3 (6%) | 8 (15%)	0.0002*** | 0.012***	0.0002***
African American/Black	35 (49%) | 29 (41%)	15 (21%) | 23 (32%)	22 (31%) | 19 (27%)		
Other	30 (77%) | 25 (64%)	6 (15%) | 8 (21%)	3 (8%) | 6 (15%)		
*Comfortable Discussing Vaccines with OHCPs*					
White	39 (75%) | 39 (75%)	7 (13%) | 7 (13%)	6 (12%) | 6 (12%)	0.0001*** | <0.0001***	0.0013***
African American/Black	25 (35%) | 22 (31%)	31 (44%) | 30 (43%)	15 (21%) | 18 (26%)		
Other	23 (59%) | 19 (49%)	6 (15%) | 8 (21%)	10 (26%) | 12 (31%)		

Abbreviation: MANOVA, multivariate analysis of variance.

a Statistical tests for each vaccine: Chi‐Squared/Fisher's Exact Test.

b Statistical tests between vaccines: MANOVA Test.

c *p*‐Value significance code: *p* < .01***, 0.01 < *p* < .05**, 0.05 < *p* < .1*.

## 4 DISCUSSION

Utilizing OHCPs as an additional resource for vaccine education and administration could help increase access to vaccines. Existing literature has discussed the dentist's role in administering vaccines with a focus on the HPV vaccines as a pathway to prevent certain head and neck cancers. While not guaranteed to prevent development of oropharyngeal or other cancers, nor to always prevent infection with HPV, vaccination has been found to be highly effective in prevention of infection and is theorized to contribute to a decrease in HPV‐positive cancers across body sites (Shapiro, 2022). Although this impact on oropharyngeal cancers is not immediately evident, as vaccinated populations age, the protective benefit of HPV prevention will become increasingly clear (Choi et al., 2022; Schuman et al., 2022). Our study expands upon previous research by investigating patient perspectives of OHCPs educating, discussing, recommending, and administering vaccines for both COVID‐19 and HPV.

In our survey, a large majority of respondents reported being previously informed that COVID‐19 vaccines could prevent severe illness caused by COVID‐19. However, only about half of respondents had prior knowledge that the HPV vaccines could prevent some forms of head and neck cancer. This emphasized a general lack of knowledge about HPV and its link to head and neck cancers, despite the rising incidence of HPV‐related OPSCCs in the United States (Centers for Disease Control and Prevention, 2021a). OHCPs should take roles in primary prevention through vaccine advocacy and secondary prevention roles through routine head and neck exams to combat this rise (Arnell et al., 2021; Chaturvedi et al., 2011).

Most participants responded in favor of having OHCPs involved in vaccine‐related efforts for COVID‐19 and HPV. Over half of respondents believed that dental providers were qualified to educate about the COVID‐19 and HPV vaccines. Large majorities also felt comfortable discussing the COVID‐19 and HPV vaccines with their dental provider. Previous studies have shown similar rates of acceptance of information on HPV and the HPV vaccines from dental providers (Dean et al., 2020; Stull et al., 2020). About half of respondents in our study were also comfortable with a dentist administering the COVID‐19 or HPV vaccines, also consistent with previous research (Dean et al., 2020). Including dental professionals in the delivery of vaccines could strengthen collaborations with medical providers and have a significant impact on access to vaccines (Serban et al., 2020). A 2022 survey found that a majority of dentists were willing to administer COVID‐19 and influenza vaccines. However, only 2% were currently administering vaccines and only one‐third were providing vaccine education to patients (Duong et al., 2022). Therefore, increasing OHCP's comfort in educating and administering vaccines may help to influence their involvement in vaccine efforts.

In general, respondents answered more favorably toward OHCPs' involvement with the COVID‐19 vaccines compared to the HPV vaccines, despite the association between HPV and oropharyngeal cancer. A higher proportion of respondents believed that dental professionals were qualified to educate about COVID‐19 over HPV vaccines. This may be attributed to heavy media coverage on COVID‐19 vaccines during the study period, while HPV vaccines have received minimal attention in the media (Gollust et al., 2016). The discrepancy in coverage, paired with reduced awareness about the risks of HPV, could explain less favorable responses to HPV‐related survey questions.

Our study identified vaccine status as a strong predictor of responses about the dentist's role in vaccine efforts. Compared to those unvaccinated against COVID‐19, patients vaccinated against COVID‐19 were more likely to answer favorably toward OHCPs' involvement in vaccine efforts. Our results found that of those previously vaccinated against COVID‐19, over 90% were comfortable discussing the COVID‐19 vaccines with a dental provider and over 80% would accept the recommendation for COVID‐19 vaccination from a dental provider. Responses from those planning to receive the COVID‐19 vaccines, compared to those not planning to receive the COVID‐19 vaccines, followed a similar trend. This mirrors other data that individuals receptive to various vaccination efforts may be more comfortable with additional vaccines. This may belie a more general lack of trust in the scientific process, in medical providers, in other figures of authority, or other factors in patients not willing to receive vaccines, while those who had accepted vaccination may be in general more receptive to these efforts. Regardless, this trend suggests that the previously vaccinated group is more receptive to incorporating dental providers in future vaccination (Nindrea et al., 2021).

Of note, in our results, a high proportion of respondents who were planning to receive the HPV vaccines and of those unsure if planning to receive the HPV vaccines answered favorably for dentist's involvement in vaccine efforts. Over 70% of those either planning to receive the HPV vaccines or those unsure if planning to receive the HPV vaccines were comfortable discussing HPV vaccines with dental providers. A majority also believed dental providers were qualified to educate about the HPV vaccines and would accept the recommendation for an HPV vaccine. Considering only about half of responders were aware that HPV was associated with head and neck cancers, it is notable that a substantial proportion of those unvaccinated against HPV were receptive to vaccine recommendations from dental providers. This may reflect the positive impact of vaccine recommendation from healthcare providers on uptake (Ylitalo et al., 2013). Individuals not yet vaccinated against HPV may therefore be an appropriate group to target for vaccine education and recommendation.

Lastly, responses were found to vary according to respondent race, with statistically significant differences between racial groups. Over 60% of survey participants identified with a racial minority group, with the largest proportion identifying as African American/Black (44%). African American/Black respondents' acceptance of dentist involvement in vaccine efforts were consistently lower than other racial groups. The COVID‐19 pandemic has highlighted long‐standing racial disparities in healthcare that likely apply to patient acceptance of both vaccines in question (Mackey et al., 2021). Further studies should examine existing racial disparities in healthcare and factors contributing to vaccine acceptance among African American/Black populations.

One avenue to expand patient access and to grow the role of OHCPs in disease prevention through vaccination is through training and vaccine administration programs in dental schools. Expanding routes to vaccination is recognized across healthcare settings as central to addressing vaccine hesitancy and to raising vaccination rates against COVID‐19 and other diseases (Shen & Tan, 2022). As patients with less advantageous circumstances are often seen in dental schools, efforts to make healthcare easily accessible and convenient may make a particular impact in this setting. In fact, expanding vaccination efforts to dental schools may help build patient trust in OHCPs, train the dental workforce, and provide avenues for care for patients who have established trust with their OHCP. This would be particularly important given the proven relationship between low trust in providers, lack of accurate information, and vaccine hesitancy (Jennings et al., 2021; Neely et al., 2022). Dental schools are established community locations equipped with all supplies and personnel needed to address any complications that may occur after vaccine administration and may serve patients already presenting for care. Creating community vaccination programs within dental schools and educating OHCPs on the administration of vaccines may serve as a modern approach to prevent disease. These programs, if established, could be tracked to determine their patient reach. Evidence of patient vaccination could also provide further data on the ability for OHCPs to contribute to vaccination efforts.

This study presents the first approximation of patient awareness and attitudes around the role of dental providers in vaccination that compared two types of vaccines and is the first to report on the role of the dentist in vaccination for COVID‐19. However, it does come with limitations, including that the survey was distributed when all COVID‐19 vaccines in the United States were authorized for Emergency Use only (April–June 2021). Survey respondents may have been reluctant to accept a vaccine under emergency approval and may have been reluctant to accept a vaccine from an OHCP when there was no precedence for this in our state. COVID‐19 vaccine recommendations also changed during the study timeframe, which may have affected patient responses. Questions about vaccination status did not collect specifics, including manufacturer or number of doses for either COVID‐19 or HPV, which may have compromised the comprehensibility of our study or the quality of results. The survey was distributed at a single academic institution in an urban area and results may therefore not reflect all populations or locations. In addition, the results may suffer from selection bias given patient recruitment solely among adults seeking dental treatment. Other limitations of survey‐based research, including recall and social acceptability bias, may also have affected our results. Finally, while our instrument was adapted from a previous survey, it was not validated.

## 5 CONCLUSIONS

Dental professionals are of central importance in the early diagnosis and treatment of oral and oropharyngeal cancers and should be at the forefront of providing primary disease prevention through HPV vaccination. However, patient acceptance of vaccines continues to be a challenge in obtaining widespread immunization. This study verified patient willingness to consider education, recommendations, and administrations of vaccines from OHCPs, including those for COVID‐19 and HPV. Involving OHCPs from diverse backgrounds, including dental students, may offer an increased level of trust within minority patient communities. Expanding the scope of dentistry to include vaccine education and administration could increase access to care, vaccine uptake, and disease prevention. Future research should examine increasing the dental providers' roles in vaccine education and administration and adding training in vaccination to the dental school curriculum.

## AUTHOR CONTRIBUTIONS

*Conceptualization, ideas; formulation or evolution of overarching research goals and aims*: Sara Steinbaum, Roopali Kulkarni, Katherine France. *Methodology development or design of methodology; creation of models*: Sara Steinbaum, Patricia Corby, Roopali Kulkarni, Katherine France. *Software programming, software development; designing computer programs; implementation of the computer code and supporting algorithms; testing of existing code components*: Patricia Corby, Katherine France. *Validation verification, whether as a part of the activity or separate, of the overall replication/reproducibility of results/experiments and other research outputs*: Patricia Corby, Katherine France. *Formal analysis application of statistical, mathematical, computational, or other formal techniques to analyze or synthesize study data*: Katherine France. *Investigation conducting a research and investigation process, specifically performing the experiments, or data/evidence collection*: Sara Steinbaum, Julia Jagannath, Lake Seymour. *Resources provision of study materials, reagents, materials, patients, laboratory samples, animals, instrumentation, computing resources, or other analysis tools*: Patricia Corby, Katherine France. *Data curation management activities to annotate (produce metadata), scrub data and maintain research data (including software code, where it is necessary for interpreting the data itself) for initial use and later reuse*: Katherine France. *Writing—original draft, preparation, creation and/or presentation of the published work, specifically writing the initial draft (including substantive translation)*: Sara Steinbaum, Julia Jagannath, Lake Seymour. *Writing—review and editing, preparation, creation and/or presentation of the published work by those from the original research group, specifically critical review, commentary or revision—including pre‐or postpublication stages*: All authors. *Visualization preparation, creation and/or presentation of the published work, specifically visualization/data presentation*: Sara Steinbaum, JagaJulia Jagannath, Lake Seymour, Roopali Kulkarni, Katherine France. *Supervision oversight and leadership responsibility for the research activity planning and execution, including mentorship external to the core team*: Patricia Corby, Roopali Kulkarni, Katherine France. *Project administration management and coordination responsibility for the research activity planning and execution*: Katherine France. *Funding acquisition, acquisition of the financial support for the project leading to this publication*: Patricia Corby, Katherine France.

## CONFLICT OF INTEREST STATEMENT

The authors declare no conflict of interest.

## Data Availability

The data that support the findings of this study are available from the corresponding author upon reasonable request.
